# Healthy lifestyles and physical fitness are associated with abdominal obesity among Latin‐American and Spanish preschool children: A cross‐cultural study

**DOI:** 10.1111/ijpo.12901

**Published:** 2022-03-01

**Authors:** Pedro Ángel Latorre‐Román, Iris Paola Guzmán‐Guzmán, Juan Antonio Párraga‐Montilla, Felipe Caamaño‐Navarrete, Jesús Salas‐Sánchez, Constanza Palomino‐Devia, Felipe Augusto Reyes‐Oyola, Cristian Álvarez, Ana de la Casa‐Pérez, Antonio J. Cardona Linares, Pedro Delgado‐Floody

**Affiliations:** ^1^ Department of Didactics of Music, Plastic and Corporal Expression University of Jaén Jaén Spain; ^2^ Faculty of Chemical‐Biological Sciences Universidad Autónoma de Guerrero Guerrero Mexico; ^3^ Faculty of Education Universidad Católica de Temuco Temuco Chile; ^4^ Universidad Autónoma de Chile Providencia Chile; ^5^ Faculty of Education Sciences Universidad de Tolima Tolima Colombia; ^6^ Department of Health Universidad de Los Lagos Osorno Chile; ^7^ Exercise and Rehabilitation Sciences Laboratory, School of Physical Therapy, Faculty of Rehabilitation Sciences Universidad Andres Bello Santiago Chile; ^8^ University Pablo Olavide Sevilla Spain; ^9^ Department of Physical Education, Sport and Recreation Universidad de La Frontera Temuco Chile

**Keywords:** cardiorespiratory fitness, children, nutritional level, obesity, physical activity

## Abstract

**Background:**

Identifying environmental factors that influence health in children are necessary to develop preventive strategies.

**Objective:**

To determine the association between the lifestyles of children (i.e., Mediterranean diet (MD), physical activity (PA), fitness and screen time (ST) with abdominal obesity (AO) of preschoolers from three Spanish‐speaking countries (Chile, Colombia and Spain) with different socioeconomic levels and Human Development Index (HDI) indicators.

**Material and Methods:**

This cross‐sectional study included 982 schoolchildren (aged 4–6 years; 56.8% girls) from Chile (*n* = 409), Colombia (*n* = 281), and Spain (*n* = 292). Body mass index (BMI), waist circumference (WC), waist‐to‐height ratio (WtHR), adherence to the MD, PA, ST and physical fitness were evaluated.

**Results:**

Spanish preschoolers reported a lower WtHR (*p* < 0.001), greater physical fitness (Z‐score) (*p* < 0.001) and higher adherence to the MD (*p* < 0.001) than their Chilean and Colombian peers. In addition, Colombian preschoolers had a better lifestyle (PA + ST) than their Chilean and Spanish peers (*p* < 0.001). Chilean preschoolers reported a higher prevalence of AO than the Spanish preschoolers (65% vs. 51.9%; *p* = 0.001).

**Conclusion:**

Lifestyle had a significant association with AO among Spanish‐speaking preschool children, with physical fitness especially being a relevant factor regardless of the country of origin. The findings of the current study may support the development of public guidelines focusing on healthy lifestyles in children to create effective plans that contribute to the early treatment of AO in preschool children.

## 1 INTRODUCTION

The prevalence of obesity among children and adolescents (aged 2–18 years) is an important public health problem.^1^ In 2020, globally, 38.9 million children under the age of 5 years old were overweight. In this public health problem, about half of all countries have experienced no progress or are worsening. Particularly, childhood obesity in preschoolers is a global phenomenon in developed and undeveloped countries where nutritional deficiencies and environmental factors from the lifestyles of parents can explain part of their children's health, but where other direct lifestyle factors, such as diet and physical activity (PA) can also play a critical role promoting greater energy expenditure.^2^ In this regard, it is noteworthy that malnutrition and lower levels of PA increase childhood obesity.^3^ Children with obesity show breathing difficulties, increased risk of fractures, hypertension, early markers of cardiovascular disease, insulin resistance, and psychological effects.^3^

It should be noted that the preschool age (2–4 years old) is very important in the development of adipose tissue, as it covers the rebound of adiposity.^4^ Especially, Abdominal obesity (AO) is associated with increased cardiovascular risk in preschoolers.^5^ Mainly, the waist‐to‐height ratio (WHtR), which is calculated as waist circumference (WC) divided by height, has gained a lot of attention such as an anthropometric index for central adiposity that allows identifying cardiometabolic risk in pre‐schoolers, children, youth and adults in different regions around de world.^6^ Although the adverse health effects of AO are well recognized in adults, this pattern of relationships remains to be determined in paediatric populations.^7^Therefore, early identification of risk factors associated with overweight/obesity in the preschool population, especially concerning lifestyles, is essential to prevent long‐term health consequences.

Although obesity is primarily the result of an imbalance between energy intake and expenditure,^8^ the explanations for AO in preschool children are various, including parental obesity (i.e., familial inheritance), screen time (ST) or sedentary behaviour, PA (particularly moderate‐to‐vigorous PA), parents education, birth weight of children,^9^ low socioeconomic status of parents, adherence to the Mediterranean diet (MD) or physical fitness, as a consequence of PA patterns.^9^, ^10^, ^11^, ^12^ It is noteworthy that changes in the global food system with caloric intake, high periods of low PA, and sedentary time are identified as the main predictors of obesity in preschool children.^4^

The food habits of children are determinant factors that influence the development of obesity.^13^ There is evidence that adherence to the MD is associated with positive effects on specific components of health,^14^ and it has an influence on health in later life.^15^ Additionally, better adherence to the MD in preschool children is associated with a lower risk of developing AO in childhood and adolescence.^12^

On the other hand, high ST has been associated with a cluster of behavioural risk factors in children,^16^ and PA has been indicated as an important factor to maintain physical health^17^ due to it being a modifiable factor.^18^, ^19^ In this regard, there is an association with low levels of moderate‐to‐vigorous PA and higher obesity status among preschool children.^8^, ^20^ Likewise, physical fitness is considered as an important health marker in early childhood and later in life.^21^, ^22^, ^23^ Moreover, it seems important to assess the ability of the respiratory systems, since negative cardiorespiratory fitness (CRF) in these stages is associated with adverse cardiovascular indicators (e.g., hypertension).^24^ In fact, the evidence have established CRF cut points to avoid cardiovascular disease risk in children and adolescents, identifying CRF as an important indicator of cardiometabolic health.^25^

Increasing levels of childhood obesity over the last half‐century indicate huge sociocultural changes, such as increased urbanization, economic growth, modernization, and globalization of food markets.^26^, ^27^ Across different societies, cultural factors show a strong influence on body size, food culture, and PA for both adults and children.^26^ Although, in the current globalized society, unhealthy behaviours can be similar in different cultures. In this regard, cross‐cultural studies may be interesting to comprehend how dissimilar environments and different cultures may influence lifestyles.^28^ Therefore, the cross‐cultural differences in lifestyle with regard to overweight and obesity are very relevant to scientists and governments, in most countries.

To our knowledge, there are no studies that have analysed the prevalence of AO in preschool children and its relationship with lifestyles through cross‐cultural analysis, particularly among Latin‐American and European populations (such as in Spain).

Therefore, this study focuses on three different countries in terms of social, economic, and environmental characteristics: Spain, Chile, and Colombia. Spain and Chile are regarded as having a very high Human Development Index (HDI),^29^ 0.904 and 0.851, respectively, and Colombia showed a high HDI (0.767). Also, the per capita gross domestic product (current US $) indicated differences between the three countries, Chile = 13231.7, Colombia = 5332.8 and Spain = 27057.2.^30^ However, these three countries have a growing problem of obesity among their child populations.^31^, ^32^, ^33^ Other local studies showed a prevalence of 50.4% and 50% of AO in Spanish and Colombian preschoolers, respectively, but up‐to‐date information is not available for the entire country.^34^, ^35^

Therefore, the purpose of this study was to determine the association between the lifestyles of children (i.e., MD, PA, and ST) with AO of preschoolers from three Spanish‐speaking countries (Chile, Colombia, and Spain) with different socioeconomic levels and HDI indicators. It is expected that the findings from this cross‐cultural study will provide practical information on the lifestyles of preschoolers and physical fitness, as well as the prevalence of AO, regarding boys and girls in the three countries.

## 2 MATERIAL AND METHODS

A priori sample size was performed using the G‐Power. The following parameters were selected for the analyses of covariance (ANCOVA): moderate effect size (w = 0.252), significance level of 0.05, a power level of 0.95, three‐group, noncentrality parameter λ =15.68, critical F = 3.032. The sample size was determined to be at least 251 participants.

This cross‐sectional study included 982 schoolchildren (4–6 years old; 56.8% girls) from Chile (*n* = 409), Colombia (*n* = 281), Spain (*n* = 292) selected for convenience from 15 schools (5 per country) of various rural and urban areas. Parents and guardians were informed about the study and provided signed written consent for participation. The investigation complied with the 2013 Declaration of Helsinki and was approved by the local ethics committee (CEIH 120215‐1, ACTAN°086_2017, ABR.19/8.TES Act).

The inclusion criteria were as follows: (i) presenting informed consent of the parents, (ii) belonging to educational centres, and (iii) being between 4 and 6 years of age. The exclusion criteria were as follow some medical condition such as some neurodevelopmental or neuromotor disability (autism, Down's syndrome), and/or the presence of some disorder associated with the cardiorespiratory and locomotor systems such as asthma, muscle injuries, and so forth.

## 3 HEALTH MARKERS

### 3.1 Physical fitness

For the evaluation of physical fitness, leg strength, cardiorespiratory fitness (CRF), velocity and handgrip strength were measured.

Lower body explosive strength was assessed by the standing long jump test (SLJT). The SLJT has been used in preschool children^36^ and consists of jumping a distance with both feet at the same time. For this, the student stands behind the jump line, and with foot separation equal to the width of their shoulders, the knees are then bent with the arms in front of the body and parallel to the ground. From this position, they swing their arms, push hard and jump as far as possible, making contact with the ground with both feet simultaneously and in a vertical position. The test was performed twice, and the best score was recorded in centimetres (cm) (between takeoff and the heel of the nearest foot at landing). The test score was the distance reached, a lower distance indicating poorer performance. Handgrip strength was used to measure upper body strength through a hand dynamometer (TKK 5101 Grip D; Takei, Tokyo, Japan). The test consists of holding a dynamometer in one hand and squeezing it as tightly as possible without allowing the dynamometer to touch the body; force is applied gradually and continuously for a maximum of 3–5 s.^37^ The test was performed twice, and the maximum score for each hand was recorded in kilograms (kg). The average of the scores achieved by the left and right hands was used in the analysis. Higher scores indicated better performance.

CRF was assessed using the 10 × 20 m test, inspired by the spatial structure of the Léger test. The 10 × 20 m test has been used in preschool children and has been validated.^38^ Materials required include a tape measure to mark the distances of the runway (20 m), two boxes, five balloons, and a stopwatch. It is a 20 m shuttle test, in which participants have to move five balloons from box A, located at one extreme, to box B, located at the opposite extreme. The total distance covered is 200 m, timed from the signal “Go” until the participant deposits the last balloon. It does not matter if the balloon does not enter the box. If the balloon is dropped during movement, the participant must take it and carry on moving. The test allows running and walking. Only one attempt is allowed. The result is recorded in seconds with one decimal place. The test score was the running time, a longer time indicating poorer performance. Finally, the 20 m sprint test (two trials with 120 seconds of recovery between trials, the best trial was registered) were performed on a flat, hard, non‐slip surface, with the start line and finish line marked. The children were motivated to run as fast as possible. The test was measured with a stopwatch to the nearest of 0.1 seconds. A lower score indicates a greater performance.

### 3.2 Abdominal obesity

Waist‐to‐height ratio (WtHR) is a simple, yet effective, a surrogate measure of AO and may be a good predictor of cardiovascular disease risk in children.^10^ Waist circumference (WC) was measured using a Seca® 201 tape measure (Hamburg, Germany) at the height of the umbilical scar. The WtHR was obtained by dividing the WC by the height in cm. To define AO a WtHR ≥0.50 was considered^39^, ^40^. The body mass (in kg) was measured using a TANITA scale, model Scale Plus UM – 028 (Tokyo, Japan); the children were weighed in their underclothes without shoes, and the height (in m) was estimated with a Seca® stadiometer, model 214 (Hamburg, Germany) that was graduated in millimetres. In addition, the body mass index (BMI) was calculated as the body mass divided by the square of the height in meters (kg/m^2^).

## 4 LIFESTYLE

Adherence to the MD was assessed by the Krece Plus test,^41^ which is a tool to assess the eating pattern and relationship with the nutritional status based on the MD. The questionnaire has 15 items, and the format assesses a set of items about the food consumed in the diet. Each item has a score of +1 or −1, depending on whether it approximates the ideal of the MD. The total points are added, and according to the score, the nutritional status is classified as follows: (i) low nutritional level: ≤5; (ii) moderate nutritional level: 6–8; and (iii) high nutritional level: ≥9. Moreover, the Krece Plus test classifies lifestyle based on the daily average of hours spent watching television or playing video games per day as ST and the hours of PA after school per week. The classification is made according to the number of hours used for each item. The total points are added, and the person is classified as having a good lifestyle (men: ≥9, women ≥8), regular lifestyle (men: 6–8, women: 5–7), or bad lifestyle (men: ≤5, women: ≤4) according to the lifestyle score.

### 4.1 Procedure

All tests were conducted in schools – in their sports facilities and classrooms, with teachers present, during recess. In two separate sessions, a team of researchers previously trained in conducting the different tests evaluated participants. During the first testing session, the anthropometrics variables were assessed. During the second testing session, physical fitness tests were recorded. Prior to the testing sessions, children performed a typical warm‐up consisting of 5 min of low‐intensity running and 5 min of general exercise (i.e., skipping, leg lifts, lateral running and front‐to‐behind arm rotations). The children also performed some familiarization trials. Each child was individually assessed. The research team conducted a demonstration. The children were motivated and encouraged to reach the best score possible in every test. The questionnaires were completed individually by the parents at home (respecting data confidentiality and clarifying any potential doubts or questions).

### 4.2 Statistical analysis

Data were analysed using SPSS, v.22.0 for Windows (SPSS Inc, Chicago, IL). The significance level was set at *p* < 0.05. Descriptive data are reported in terms of means and standard deviations (SDs). Tests of normal distribution and homogeneity (Kolmogorov–Smirnov and Levene's, respectively) were conducted on all data before analysis. Differences between groups were determined using analysis of variance (ANOVA) (adjusted by age and sex) with post hoc analysis adjusted by the Bonferroni test. The association of lifestyle with AO was evaluated across the β coefficient using the regression logistic model (adjusted by age and sex) or by Mantel–Haenszel test in dichotomy variables. The inclusion of odds ratios (ORs) with 95% confidence intervals (CIs) were used.

## 5 RESULTS

Sociodemographic characteristics indicated that Chilean and Colombian children had a lower socioeconomic level than Spanish children (43.3% vs. 37.0% and 11.0%, respectively; *p* < 0.001); in addition, 90.4% of the parents of Spanish children were married, with significant differences (*p* < 0.001) concerning Chilean and Colombian children, who registered percentages of 45.5% and 45.6%, respectively. No significant differences were found in educational level (*p* = 0.279).

Table 1 shows anthropometric parameters, physical fitness and lifestyle according to country. Spanish preschoolers reported a lower WtHR (*p* < 0.001) than their Chilean and Colombian peers. Likewise, regarding fitness, the Spanish preschoolers reported better physical fitness (Z‐score) than their Chilean and Colombian peers (*p* < 0.001). In addition, Colombian preschoolers had a better lifestyle (PA + ST) than their Chilean and Spanish peers (*p* < 0.001). However, Spanish preschoolers reported higher adherence to the MD than their Latin‐American peers (*p* < 0.001).

**TABLE 1:** Anthropometric, physical fitness and nutritional status in preschool children according to country

Parameters	Chile (*n* = 409)	Colombia (*n* = 281)	Spain (*n* = 292)	*p* value
Age (years)	5.13 (0.57)	5.03 (0.65)	4.97 (0.50)	0.001
% boys	45.0	44.8	39.0	0.235
Anthropometric				
Body mass (kg)	19.87 (4.31)a	19.80 (3.78)a,b	20.29 (3.61)b	0.037
Size (m)	1.13 (0.09)	1.12 (0.07)	1.12 (0.06)	0.226
BMI (kg/m^2^)	15.64 (3.60)a	15.71 (2.53)a,b	16.12 (2.49)b	0.033
BMI Z‐Score	−0.05 (1.19)a	−0.03 (0.84)a,b	0.10 (0.83)b	0.031
WC (cm)	58.85 (4.79)a	56 0.75 (4.79)b	55.39 (5.92)c	<0.001
Weight status (Z‐score)	0.12 (0.79)a	−0.06 (0.61)b	−0.12 (0.79)b	<0.001
WtHR(WC/height)	0.52 (0.06)a	0.51 (0.05)b	0.49 (0.05)c	<0.001
Fitness				
Test 10 × 20 (s)	78.06 (10.89)a	85.57 (16.82)b	78.28 (17.54)a	<0.001
SLJT (cm)	82.18 (20.99)	80.78 (22.65)	82.35 (21.68)	0.608
Speed 20 m (s)	6.11 (0.93)a	6.19 (0.97)a	5.98 (1.11)b	0.011
Handgrip strength (kg)	5.29 (1.87)a	6.61 (3.93)b	6.49 (2.15)b	<0.001
Physical fitness (Z‐score)	0.03 (0.48)a	0.07 (0.58)a	−0.11 (0.61)b	<0.001
Lifestyle				
Lifestyle PA + ST (0–10)	4.73 (2.48)a	5.21 (1.19)b	4.36 (2.02)a	<0.001
MD adherence (0–10)	4.08 (3.81)a	3.80 (2.90)a	6.39 (2.00)b	<0.001

*Note*: Different letter subscripts indicate significant differences (*p* < 0.05).

Abbreviations: BMI, body max index; MD, Mediterranean diet; PA, Physical activity; SLJT, standing long jump test; ST, screen time; WC, waist circumference; WtHR, waist‐to‐height ratio.

**FIGURE 1:**
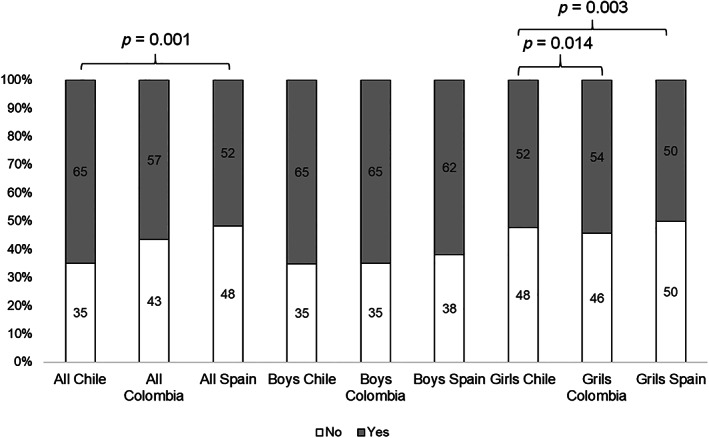
Frequency of abdominal obesity children according country (Panel A) and comparison according sex (Panel B) Chile‐Colombia‐Spain

Figure 1 shows the frequency of AO according to country (Chile, Colombia and Spain). Among all participants, Chilean preschoolers reported a higher prevalence of AO than the Spanish preschoolers (65% vs. 51.9%; *p* = 0.001). Regarding sex differences, female Chilean preschoolers reported a higher prevalence of AO than the Colombian and Spanish populations (64.9% vs. 52.3% and 50.3%; *p* = 0.014 and *p* = 0.003, respectively).

Table 2 shows lifestyle factors associated with AO in children. Physical fitness (Z‐score) and bad lifestyle were risk factor of AO in Chilean (OR = 2.103, *p* = 0.001 and OR =  2.081, *p* = 0.001, respectively). Low nutritional level (i.e., high adherence to the MD) was a protective factor of AO in Colombian children (OR = 0.553, *p* = 0.026). Finally, the speed component of physical fitness was a protective factor of AO in Colombian and Spanish children (OR = 0.765, *p* = 0.043 and OR = 0.783, *p* = 0.034 respectively).

**TABLE 2:** Lifestyle factors associated with abdominal obesity in preschoolers according to country

	Chile	Colombia	Spain
	OR (CI95%) *p* Value	OR (CI95%) *p* Value	OR (CI95%) *p* Value
Test 10x20 (s)	**1.056 (1.032**–**1.079), *p* < 0.001**	1.013 (0.997–1.029), *p* = 0.110	0.998 (0.985–1.012), *p* = 0.779
SLJT (cm)	0.991(0.981–1.000), *p* = 0.061	1.008 (0.997–1.019), *p* = 0.168	0.998 (0.987–1.009), *p* = 0.722
Speed 20 m (s)	1.167 (0.931–1.462), *p* = 0.180	**0.765 (0.590–0.991), *p* = 0.043**	**0.783 (0.624–0.982), *p* = 0.034**
Handgrip strength (kg)	1.014 (0.909–1.131), *p* = 0.802	1.022 (0.960–1.088), *p* = 0.500	0.978 (0.871–1.098), *p* = 0.704
Physical fitness (Z‐score)	**2.103 (1.354–3.266)**, ***p* = 0.001**	0.775 (0.506–1.189), *p* = 0.243	0.832 (0.545–1.271), *p* = 0.395
ST (3‐5 h vs.0–2 h)	**2.210 (1.420–3.439)**, ***p* < 0.001**	1.286 (0.800–2.065), *p* = 0.299	0.708 (0.446–1.125), *p* = 0.144
PA (0–2 h vs. 3‐5 h)	**2.294 (1.389–3.789)**, ***p* = 0.001**	0.783 (0.488–1.256), *p* = 0.310	0.765 (0.476–1.229), *p* = 0.268
Bad Lifestyle (PA + ST)	**2.081 (1.358**–**3.191), *p* = 0.001**	1.190 (0.653–2.169), *p* = 0.569	0.846 (0.526–1.361), *p* = 0.490
Low nutritional level	0.780 (0.518–1.177), *p* = 0.237	**0.553 (0.328–0.932), *p* = 0.026**	0.849 (0.519–1.387), *p* = 0.513

*Note*: The data shown represent OR (95% CI), adjusted by age and sex. Values of *p* < 0.05 were considered statistically significant (in bold).

Abbreviations: PA, physical activity; SLJT, standing long jump test; ST, screen time.

## 6 DISCUSSION

The purpose of this study was to determine the association between the lifestyles of children (i.e., MD, PA, and ST) with AO in preschoolers from three Spanish‐speaking countries with different socioeconomic levels and HDI indicators. To our knowledge, our study is the first to examine these relationships. The main results were as follows: (1) Chilean children showed a higher prevalence of AO, with significant differences with Colombian and Spanish girls; (2) Chilean children had lower levels of physical fitness than Colombian and Spanish children; (3) In particular, Spanish preschoolers reported a lower WtHR, a higher adherence to the MD and greater physical fitness than their Colombian and Chilean peers; however, Colombian children showed better lifestyles than their Spanish and Chilean peers.

Physical fitness was the common factor between countries to predict AO. Overall, the current findings add to a growing body of literature in children and adolescents about the relationship between physical fitness and AO,^42^, ^43^, ^44^ particularly in preschool children.^45^, ^46^ Given that fitness level is a potential biomarker of health from an early age, improvements in physical fitness could be important for the health of preschoolers.^47^ Therefore, replacing sedentary time and/or low PA with moderate and vigorous PA in children and adolescents is favourably associated with most markers of cardiometabolic risk.^48^ Likewise, stimulating higher intensity PA and reducing sedentary behaviour at a young age may have long‐term beneficial effects on body composition and physical fitness in later childhood.^49^

In addition, overall bad lifestyle and physical fitness (Z‐score) were risk factors for AO only in the Chilean population. According to this, another study conducted on Chilean schoolchildren reported that a bad lifestyle was associated with low physical fitness related to health; additionally, schoolchildren with a good lifestyle had better VO_2_max.^50^ In the same way, a bad lifestyle (i.e., ST) was inversely related to the motor skills of preschool children.^51^ Conversely, a good lifestyle (i.e., objective measured PA) was positively associated with physical fitness in Chinese preschoolers.^52^ In this sense, physical inactivity is recognized as a determinant of low physical fitness in preschoolers.^53^ In the present study, 0–2 h versus 3–5 h of PA per week and ST (3–5 h vs.0–2 h) are risk factors for AO in the Chilean population.

In turn, is remarkable that higher adherence to a healthy lifestyle (i.e., PA, sleep time, television time, plant‐based foods) at age 4 years decreased the risk of overweight, obesity, and abdominal obesity at age 7 years in Spanish children.^54^ Recently, Musálek et al. show that normal weight obesity seems to develop from early childhood and is related to low physical fitness and deficits in eating habits, which might inhibit the natural necessity for PA from the pre‐school stage.^55^

Moreover, in Spanish preschool children, a previous study found that higher adherence to the MD and higher CRF were associated with lower WtHR.^56^ In this regard, the current study indicates that Spanish preschoolers displayed high adherence to the MD, high physical fitness, and a lower WtHR than their Chilean and Colombian peers. Strangely low adherence to the MD, was a protective factor of AO in the Colombian population. Conversely,there are several studies in children on the relationship between obesity and eating habits.^57^, ^58^, ^59^ Children with high adherence to the MD were especially less likely to be overweight or have obesity.^60^ In this regard, the nutritional quality of the foods consumed and eating behaviours are related since diets rich in animal products, protein, fat, sugar, and salt were associated with an excess of fat mass.^4^

On the other hand, Chilean children displayed worse results of AO and several factors could have explained this finding. The ST (3–5 h/day) and PA after school (0–2 h/week) reported a positive association with AO in Chilean children. According to this, it has been reported that more ST increases AO and BMI in preschoolers.^61^ In this regard, Mota et al. showed that those preschoolers exhibiting more ST are more likely to have more AO.^62^ Moreover, Matlosz et al. found that a bad lifestyle (i.e., ST over 120 min per day together with participating less than once a week in at least 60 min of MPVA) was associated with AO and excess adiposity in Polish preschoolers.^9^ Likewise, a longitudinal study showed that more daily ST was associated with a higher BMI Z‐Score and WtHR at 6 years of age. Additionally, this study concluded that excessive ST during the preschool period is a risk factor for increased BMI Z‐Score.^63^ Similarly, another longitudinal study demonstrated that an unhealthy lifestyle, such as bad food habits and high ST, was associated with high body fat in preschool girls.^64^

The limitations of the present study include those inherent to its transversal character, likewise, the sample was not randomized. Another limitation would be the self‐reporting concerning the preschool the PA and ST of children, which could mean that these data are underestimated or overestimated by parents. In addition, other factors, such as national or local strategies to promote PA, should be analysed because preschool‐age children are a population that is not taken into account in PA programmes. Finally, the PA of parents was not analysed. However, this study contributed to exploring several variables that affect children's health and have contributed to a better understanding of the serious problem of bad lifestyle and AO in preschoolers in an intercontinental study. We project the need to investigate possible longitudinal effects clarify the direction of the associations and carry out interventions in children's lifestyles.

From a practical point of view and considering the lack of reference values for AO and WtHR in preschool children through a cross‐cultural study, the values obtained in this study can be used as a ‘warning signal’, where it would be necessary to carry out additional tests to identify possible health problems early. In turn, in this study, we evidenced that low levels of PA and poor physical fitness, and adherence to the MD are risk and protective factors respectively of AO in preschool children. Therefore, nutrition education programs and the improvement of children's PA levels must be incorporated into the school environment.

## 7 CONCLUSION

Lifestyle had a significant association with AO among Spanish‐speaking preschool children, with low physical fitness especially being a risk factor for AO. To sum up, Chilean children exhibit the worst prevalence of AO, which was fundamentally influenced by low physical fitness and bad lifestyles. The findings of the current study may support the development of public guidelines focusing on healthy lifestyles in children to create effective plans that contribute to the early treatment of AO in pre‐schoolers.
